# Potential Peripheral Biomarkers for the Diagnosis of Alzheimer's Disease

**DOI:** 10.4061/2011/572495

**Published:** 2011-10-23

**Authors:** Seema Patel, Raj J. Shah, Paul Coleman, Marwan Sabbagh

**Affiliations:** Banner Sun Health Research Institute, Sun City, AZ 85351, USA

## Abstract

Advances in the discovery of a peripheral biomarker for the diagnosis of Alzheimer's would provide a way to better detect the onset of this debilitating disease in a manner that is both noninvasive and universally available. This paper examines the current approaches that are being used to discover potential biomarker candidates available in the periphery. The search for a peripheral biomarker that could be utilized diagnostically has resulted in an extensive amount of studies that employ several biological approaches, including the assessment of tissues, genomics, proteomics, epigenetics, and metabolomics. Although a definitive biomarker has yet to be confirmed, advances in the understanding of the mechanisms of the disease and major susceptibility factors have been uncovered and reveal promising possibilities for the future discovery of a useful biomarker.

## 1. Introduction

The incidence of Alzheimer's disease (AD), the most prevalent form of dementia seen in the elderly population, is expected to increase exponentially over the next ten years. With the pervasive nature of such a debilitating disease, extensive research has focused on potential peripheral biomarkers and how they could be utilized to diagnose and monitor the progress of Alzheimer's disease. There are two types of biomarkers: state markers and stage markers. A state marker denotes the severity of the disease in the individual. As the amount of a certain state marker increases, the severity of the disease in the individual increases. Stage markers indicate how far the disease has progressed within the individual. If an individual has a certain degenerative disease, then a state and stage marker will be present [1]. According to the Consensus Report of the Working Group on Molecularand Biochemical Markers of Alzheimer Disease, a biomarker must adhere to certain basic requirements, including the ability to reflect AD pathology and differentiate it from other dementia with an 80% sensitivity, be reliable and reproducible, be easy to perform and analyze, and remain relatively inexpensive [2]. Currently, the most effective methods for ascertaining the diagnosis of AD is limited to imaging technology (e.g., MRI and PET) and the analysis of cerebrospinal fluid (CSF), which requires lumbar puncture [3]. Ideally, both technologies have good specificity and sensitivity but have limitations of expense and risks associated with invasive procedures.

The discovery of a well-established peripheral biomarker that is easily accessible and cost effective is of primary importance when considering the prevalence of this disease. The necessity for a biomarker for AD in blood is so high because of the disadvantages of the status quo. The above consideration suggests the need for a diagnostic biomarker able to detect disease prior to symptomatic onset. In order to achieve this goal, such a diagnostic biomarker needs to be one that could be part of a routine physical exam, as is currently the case for lipid profile. This implies a biomarker that could be obtained in any physician's office or specimen collecting station with minimal invasiveness at reasonable cost and time demand. In order to be adopted for wide use, the biomarker has to have demonstrated consistency in a large number of persons from a variety of populations. Current imaging and CSF biomarker studies satisfy the criteria of having been established in a large number of persons and in a variety of populations. These studies have served to establish the important principle of feasibility of early detection. However, these classes of biomarker do not satisfy the criteria of minimal invasiveness, reasonable cost, or minimal time demand. These considerations lead to a strong recommendation of the need for an effective biomarker of AD. The criteria of minimal invasiveness, minimal time by the physician, and reasonable cost argue for an easily obtained sample of peripheral tissue that could then be analyzed either in the office or at a central location. 

Diagnosis of AD is made mainly from clinical testing, and currently, there is no completely accurate test for diagnosing AD. Obtaining CSF from elderly individuals on repeated occasions is no easy task. Blood is very easy to obtain, and since CSF is absorbed into the blood every day, plasma can supply numerous biomarkers for AD 4. Therefore, finding a peripheral biomarker that uses easily collected samples (e.g., plasma, blood, saliva, and urine) would be doubly advantageous because of its relatively noninvasive procedure and ability to provide an accurate diagnosis. The search to find a peripheral biomarker that could serve as a definitive diagnostic tool that is universally available has created a vast body of studies that spans over several different biological approaches. In this paper, we will examine the current status of potential peripheral biomarkers of Alzheimer's disease, evaluating each from the perspective of being minimally invasive, easily obtainable with a minimal time requirement and reasonable cost. We will further evaluate each biomarker from the perspectives of the ability to detect already diagnosed disease and ability to predict future disease.

## 2. Evaluable Tissues

The widespread incidence of Alzheimer's has seemingly no pattern of “onset.” Because up to 98% of Alzheimer cases are sporadic, it is crucial to identify potential biomarkers from assessable tissues that could diagnose AD on an individual basis [4]. The utility of such an approach is the availability of bodily samples that could provide a noninvasive and rather inexpensive process for diagnostic determination, such as a swab of saliva, a simple blood sample, or a urine test.

The utilization of saliva as a biological marker of AD has been examined and led to the possibility of its utility in diagnosing early onset forms of the disease and differentiating AD type dementia from other forms of neurodegenerative illnesses. Biopsies of the salivary gland can produce significant findings for Alzheimer's, since salivary epithelial cells express amyloid precursor protein and A*β*. Also, it is important to note that changes in the cerebrospinal fluid may perhaps be reflected in the saliva [5]. One study compared a group of individuals with AD to a group of controls matched for age and sex as well as individuals with Parkinson's disease. The findings uncovered that there was a small, but still statistically relevant, increase of A*β*42 in patients with mild AD [5]. It should be noted that there was no noticeable change in the A*β*42 levels of either the controls of Parkinson's patients, which would indicate that the salivary levels of A*β*42 could be used to distinguish AD from other forms of dementia. Studies also suggest there is a connection between the salivary acetylcholinesterase enzyme (AChE) and AD, since it is already established that a decrease in central cholinergic activity is a noteworthy aspect of the disease biochemistry [6]. During early stages, the cholinergic neurons primarily undergo degeneration and result in a notable decrease in acetylcholine. One study revealed that in patients with AD, AChE activity was appreciably lower than in their age-matched counterparts, suggesting that salivary levels of cholinergic activity could be a biomarker [7]. The changes in salivary AChE activity appear to parallel the AD-associated decrease in brain cholinergic activity [7]. In a study done by Sayer et al., subjects receiving treatment with AChE inhibitors were classified based on whether they responded cognitively to the AChE treatment. They found a significant difference in AChE levels between those who did not respond to the treatment and their controls [7]. While these studies demonstrate the possible fronts through which a useful biomarker can be found, there have yet to be conclusive results verifying the diagnostic value of acetylcholinesterase levels and whether peripheral salivary markers truly reflect changes in cerebrospinal fluid. 

The use of blood as a tissue to yield potential biomarkers of AD has both its advantages as well as challenges. The most prominent challenge to determining the accuracy of a blood biomarker is in establishing a correlation between brain changes and dectecting those changes in blood [8]. Despite the difficulties presented by a blood-brain barrier, the possibility of a blood protein signature and notable alterations in blood-based proteins may present biomarkers that could be used to predict and monitor disease progression. Along with the ease of accessibility of blood, there is the additional advantage of the multiple tissues present in blood, namely, plasma, serum, and its cellular components (e.g., reds cells, white cells, and platelets). 

The use of plasma-based proteins offers some promising risk analysis tools. Plasma is the liquid portion of blood that suspends cells such as erythrocytes, leukocytes, and thrombocytes and proves to be an ideal fluid for biomarker inquiry due to its universal availability. Plasma can be isolated from blood by using an anticoagulant and centrifuging the sample at low speeds. It contains thousands of proteins that reflect the physiological occurrences in the body and affect the brain from the periphery as well as those proteins that are exported from the brain. Several studies have been produced to demonstrate that telomere length in peripheral blood cells are a potential marker for AD, but the relationship between peripheral blood leukocyte telomere length and the proliferation of AD pathogenesis remains unclear. In one study, the telomere length of peripheral blood leukocytes was compared to the telomere length in the cerebellum [9]. Telomere length in the cerebellum was not indicative of inherited telomere length as a determinant of AD susceptibility; rather, acquired shortening of peripheral blood leukocyte telomere lengths could be seen as an indication of chronic stress, supporting an underlying correlation between leukocyte telomere length and risk for developing AD.

Urine samples can be considered as a means of diagnosing AD through noninvasive procedures. Evaluating the proteins in urine for AD could help physicians inform patients of their prognosis. Unfortunately, researchers have attempted to develop an AD biomarker sensitive enough to be used on urine, but no such reliable and reproducible biomarker has been found to date. Early evidence showed increased concentrations of NTP in the urine of AD patients, generating immense interest among other fellow biomarker researchers. However, attempts to commercialize the test were unsuccessful, because the validity of the test was questioned because of lack of reproducibility. Another protein called the pancreatic exocrine protein, also known as pancreatic thread protein (PTP), contained a fibrillary structure that resembled fibrils located in neuritic plaques in the brains of AD patients [10]. These researchers observed extensive amounts of PTP immunoreactivity in the brains of AD patients. The study found a substantial concentration of NTP in the CSF of AD patients compared to their controls [10]. As researchers tried to reproduce the findings of this study, they found that PTP was 40 times higher in serum than in CSF. Furthermore, PTP immunoreactivity in CSF paralleled with the CSF/serum albumin ratio, which suggests that NTP in CSF is actually PTP from serum [10]. 

In particular, the AD-associated neuronal thread protein (AD7c-NTP) has been of interest as a biomarker due to its ability to reflect significant irregularities in cellular function [9]. Dementia of the AD type is symptomatic of cell loss that is caused by multiple mechanisms that involve apoptosis and abnormal mitochondrial function. The AD7c-NTP gene codes for a protein associated with causing apoptosis and therefore, the overexpression of the gene could lead to the cell loss seen in the early stages of the disease. Higher levels of AD7c-NTP can be seen in the urine of patients experiencing early AD and can even offer insight onto the severity of the dementia. In clinical uses, AD7c-NTP has been shown to be a very useful biomarker with more than 90% sensitivity for the early detection of AD [11].

To the contrary, there is much speculation about the true utility of the NTP and AD7c-NTP. The nucleotide sequence of AD7c-NTP does not share any resemblance with a pancreatic thread protein, insinuating that these two genes code for completely different proteins. Additionally, when the DNA sequence of AD7c-NTP was compared to chimpanzee and human genomes, various amounts of differences were identified. Coincidentally, these discrepancies were discovered in places where the human and chimpanzee genome were completely identical [10].

## 3. Genomics

The use of genomic technologies is valuable in identifying potential biomarkers in several neurological diseases, including Alzheimer's, and promises to provide important insight for the future in terms of personalized diagnosis and treatment based on an individuals' predisposition to a particular condition. As the genetic analysis of individuals uncovers heritable risk factors, genomic technologies will lead to a better understanding of the protein products and mechanistic pathways associated with the proliferation of the disease.

One of the ways to finding potentially useful diagnostic biomarkers is through genomics and the human genome, which allows us to better understand disease and the manner in which it proliferates. Mendelian genetic approaches have limited utility in identifying neurodegenerative afflictions like AD, because familial cases account for a rather small amount of those afflicted. It is more important to discover biomarkers that can help explain the more common sporadic cases of the disease. Among the inheritable aspects of Alzheimer's, apolipoprotein *ε*4 has been established to have a particularly strong correlation to the development of the disease. While single allele differences are rarely able to confer an accurate indication for risk of disease development, Alzheimer's disease could be an exception. Entire genome single-nucleotide polymorphism studies have been conducted, and they confirm that the ApoE locus is able to indicate, to a certain extent, the genetic susceptibility to developing AD [12, 13]. The usefulness of genome-wide association studies primarily lies in their ability to reveal susceptibility genes of a disease by uncovering DNA variants in a large-scale analysis of the human genome. Based on replication in a large number of studies, the only firmly established genetic susceptibility factor for Alzheimer disease is the *ε*4 allele of ApoE [14]. While genome-wide association studies have proven to be useful in detecting variations in DNA that can potentially be linked to the heritability of this disease, they cannot provide the biological basis of the disease.

ApoE is a lipid transport protein that is encoded in a single gene and exists as three different isoforms. Based on the traditional principles of inheritance, the dosage of the ApoE alleles, divided into *ε*2, *ε*3, and *ε*4, is strongly related to the risk for dementia of the Alzheimer's type at an increasing frequency with the increase of *ε*4 alleles [15]. Although the Apo E *ε*4 allele accounts for only 14% of the general population, approximately 50% of Alzheimer's patients carried the *ε*4 allele [16]. Both Carriers and noncarriers of the *ε*4 allele develop Alzheimer's, evidence suggests that those without the allele are more likely to show signs of the disease later [17]. As a key component of very low-density lipoproteins that is required for cholesterol transport both centrally and in the periphery, the role of ApoE *ε*4 in AD has been related to its effect on the metabolism of cholesterol [18]. Studies of ApoE polymorphisms demonstrate that the *ε*4 allele correlates with an elevation in total and low-density cholesterol levels and could, therefore, play a negative role in AD [19]. Conversely, it is suggested that the ApoE *ε*2 plays a protective role in the development of AD, lowering risk, and delaying the onset of the disease [20]. The protective ability of *ε*2 can be attributed to the opposite effect on cholesterol metabolism it has compared to the *ε*4 allele, namely lower levels of total and low-density lipoprotein cholesterol [21]. 

The different isoforms are also hypothesized to have varying effects on amyloid plaque formation and metabolism. The *ε*3 isoform can have an increased binding affinity to A*β* peptides, thereby allowing for the clearance of A*β* and prevention of neurotoxic plaque formation [22]. It is further hypothesized that the neurotoxicity of the A*β* peptides further contributes to neurodegeneration in the *ε*4 isoform. While ApoE *ε*3 is capable of protecting cells from H_2_O_2_-induced oxidative stress, the *ε*4 isoform is not as successful at this task [23]. There are several mechanistic explanations for the role of ApoE isoforms in relation to A*β* metabolism and the formation of AD plaques, but a conclusive role requires further studies to be firmly established.

Mechanistically, ApoE4 may be responsible for accelerating the degeneration of neurons, and thereby damaging synaptic stability and causing an earlier onset of dementia. One of the key contributing factors of ApoE on the development of neurological diseases like AD is linked to the manner in which it contributes to neuronal repair, remodeling, and protection. Deleterious insults to neurons could be a result of oxidative stress, ischemia, inflammation, or other stressors associated with aging. ApoE is a contributing factor to the repair of neurons through its lipid transport function. While ApoE *ε*2 and *ε*3 are effective in this process of neuronal cell maintenance, *ε*4 has been observed to be less efficient in this role [24]. This further exacerbates the cognitive decline seen in AD by affecting neuronal connections. Although ApoE isoforms have a definite and striking effect on the clearance of amyloid-*β* and cytoskeleton stability, there are no unified explanations for the manner in which ApoE4 specifically causes a notably increased risk for AD. 

Among the other biomarker candidates that have been uncovered using genomic techniques, growth factor receptor-bound associated binding protein 2 (GAB2) alleles have been shown to have an impact on AD risk for ApoE4 carriers. The GAB2 protein is involved in several important signaling pathways that could be linked to disease proliferation if interference with the expression of GAB2 were to occur, as evidenced by the elevated levels of GAB2 in at-risk neurons and GAB2 proteins found in neurofibrillary tangles [25]. One study examined the possibility of GAB2 as a modifying factor for Alzheimer's risk in ApoE4 carriers and determined a significant correlation between multiple single-nucleotide polymorphisms (SNPs) and an increased risk of disease for *ε*4 allele carriers. The study utilized a genome-wide analysis of 502,627 SNPs to characterize and determine susceptibility genes for the onset of AD. Through their surveys of these single-nucleotide polymorphisms, the researchers determined an association of AD with six single-nucleotide polymorphisms found in the GAB2 gene as well as a shared haplotype that encompassed the GAB2 gene. Additionally, it was determined that interference with the expression of normal GAB2 led to an increase in tau phosphorylation, which is typically observed in individuals with AD. Another study further investigated the hypothesis that normally GAB2 protein is associated with reducing tau phosphorylation and the formation of neurofibrillary tangles and that an isoform of the protein could play a part in increasing the susceptibility to phosphorylated tau in at-risk individuals [26]. Further replication of both studies is required, but it does provide important insight into the possibilities to better understand the pathogenesis of this disease that could later contribute to diagnosis and treatment.

Another valuable genomics marker the ApoE gene indicated that a set of single-nucleotide polymorphisms in TOMM40, which is located approximately 15 Kb upstream of ApoE, revealed a linkage disequilibrium in connection with the E4 allele and demonstrated a significant association to increased AD risk [27]. This particular gene is responsible for the formation of an essential mitochondrial membrane protein that plays an active role in protein transport. Since aberrations in mitochondrial structure or causes for oxidative stress to the mitochondria are linked to an increase of AD risk, it would be plausible to propose the TOMM40 as a genetic indicator of risk [28]. The important genetic finding associated with TOMM40 predicts the onset of AD based on the variable length of deoxythymidine homopolymer (poly-T) on the gene. Due to the high linkage disequilibrium between TOMM40 and ApoE that demonstrates an evolutionary relationship between the two genes, it is possible to note that specific variants of TOMM40 are closely associated with each of the ApoE alleles [29]. The very long poly-T variants are categorized as high-risk alleles and are linked to ApoE *ε*4 alleles 98% of the time, whereas the ApoE *ε*3 variants are subdivided into either very long or very short. While the *ε*3 allele was supposedly neutral for AD development, it is more likely the heterogeneity of AD age-of-onset seen in the *ε*3 population was a result of a linkage of the allele to both very long or very short poly-T variants [28]. The variable length of TOMM40 is, therefore, a candidate for helping predict the onset of AD and has the potential to be a clinical diagnostic tool. Another significant finding seen through a genome-wide association study was that overlapping or linked single-nucleotide polymorphisms across the TOMM40 and ApoE region showed a significant association with cases of sporadic AD [30]. These finding warrant further investigation to determine the utility of the TOMM40 region as a biomarker of AD.

The results finding both GAB2 and TOMM40 to be possible genetic indicators of AD require further study and confirmation; however, the prospects of genotypic analysis using single-nucleotide polymorphisms at multiple gene loci provides exciting possibilities for the determination of diagnostic risk analysis for this and other diseases.

## 4. Proteomics and Proteins

Another approach towards uncovering a potential diagnostic biomarker for AD relies on a large-scale analysis of proteins and protein structure that could be used to indicate risk of cognitive decline. Proteomics involves two main steps: (1) separate the proteins using multiplex assays and (2) identify the protein and its origin. The search for novel biomarkers that can be used diagnostically at early stages of AD have looked towards proteomic technologies to determine if there are proteins that can predict disease as well as monitor progression and response to treatments. Many proteomic studies have been coordinated in order to accurately diagnose AD but none have emerged as the definitive method or cluster of identifiable proteins despite early encouraging results [31]. 

Recent proteomic analysis has found that Alzheimer's patients have a dysfunctional ubiquitin carboxyl-terminal hydrolase system. The main purpose of this system, which contains ubiquitin proteasome, is to destroy misfolded proteins. Ubiquitin proteasome is a protein that safeguards other proteins from unwanted interaction between proteins. In AD patients, disfigured proteins overwhelm the ubiquitin system and leads to the amassing of many abnormal proteins in the system. Recent studies claim that the ubiquitin proteosome is a target of protein oxidation in AD patients, creating a connection between oxidative stress and Alzheimer's in patients [32]. 

Through studies of AD patients, researchers were able to find high levels (up to 10x the normal amount) of Glial fibrillary acidic protein (GFAP) in AD patients. Astrocyte cells of the CNS and has many important functions, including cell communication and mitosis. The increased levels of GFAP in AD brains (?) means that the pathway is overcompensating for its lack of influence [32].

Among the more prominently studied biomarkers for AD risk are plasma levels of A*β*42 and A*β*40, but they have not emerged to have a definite value as a predictive tool [33]. The analysis of plasma A*β*40 and 42 levels offers a noninvasive and inexpensive biomarker, since a key pathological characteristic of Alzheimer's is A*β* deposition in senile plaques. Research has indicated that increased levels of tau and phospho-tau and decreased levels of A*β*42 can accurately indicate individuals with AD in CSF [1]. If proven to be a reliable indicator of mild cognitive impairment and AD, plasma levels of A*β*40 and A*β*42 and the ratio of A*β*42/A*β*40 could be a valuable biomarker. Mutations of the amyloid precursor protein (APP), which produces Amyloid *β* protein,can result in an increase of A*β*42 and A*β*40 in patients prior to the onset of the disease [16]. In all AD patients, regardless of APP mutation, A*β* is found to collect and form deposits in the brain that lead to the creation of senile plaques. Since its role has been extensively studied and is intrinsically linked with AD, there have been therapeutic efforts to interfere with the production of A*β* and disband accumulated amyloid deposits. Due to the different cell types that are capable of producing A*β*, it is hard to establish which cells are most actively contributing to circulating plasma or the pathways of interchange of amyloid between the brain and the periphery [4]. While patients with AD certainly had increased A*β* plasma levels in the brain and skeletal muscles, it is not yet possible to consider A*β* a biomarker, since the pathways of the protein and its dispersion and uptake have yet to be fully understood.

There have been several noteworthy attempts to validate plasma A*β* as a biomarker of AD, but questions as to their reproducibility hinder verification of their ability to accurately diagnose disease. Autopsy confirmed reports illustrate the prevalence of A*β*42 deposits in AD patients as either having it be the only form of amyloid *β* protein deposited, being the major form, or simply having large levels of both A*β*42 and 40 deposited. One study indicated that while patients who have an elevated A*β*42 levels in the plasma are more at risk for developing AD, after the onset of the disease A*β* plasma levels actually decline to possibly reflect the compartmentalization of A*β* peptides in the brain [34]. Another recent longitudinal study demonstrated that low plasma levels of A*β*40 and 42 had a correlation to a rapid decline in cognition. The hypothesis to support this observation was that an increased deposition of A*β* in the brain would be reflected in a lower A*β* plasma level [35]. While these studies help explain and predict the rapid cognitive decline that is seen in the disease, it does not really elucidate the manner in which plasma levels of A*β* can affect AD risk and development that would indicate its use as a biomarker. 

A case-cohort study determined that a combination of a high base-line level of A*β*40 and low base-line concentrations of A*β*42 seemed to correlate with a higher risk of developing dementia [36]. Another study identified no individual correlation between either baseline A*β*40 or A*β*42 with a transition from mild cognitive impairment to AD, but the ratio of A*β*42/A*β*40 did demonstrate a relationship with conversion to AD [33]. The longitudinal study demonstrated that a lower A*β*42/A*β*40 ratio was indicative of a greater decline in patient cognition. Unfortunately, conflicting results and the lack of reproducibility in study findings have made it difficult to determine the exact role of A*β* in the determination of AD diagnosis.

While amyloid *β* protein has undergone extensive study in its association with AD risk, there are several other proteins in the plasma that have been analyzed as potential biomarkers of the disease. Among the more prominently CSF-based proteins studied in association with AD is tau because of the hyperphosphorylation and aggregation of tau protein that is characteristic of the disease. Tau is a state marker that is located in neuronal axons. Because tau is a state marker, increased concentrations of tau in individuals typically means a higher severity of neuronal degeneration [1]. An increase in tau in AD patients has been discovered in many different studies. However, other dementias, such as vascular dementia, can lead to an increase in tau as well. For these reasons, tau cannot be the sole biomarker for AD, because an increase in tau points to a number of different diseases [1]. There have yet to be any significant studies investigating a blood-based analysis of this particular protein and its potential as a peripheral biomarker, but other plasma proteins have been revealed to be of interest. Studies using the proteomics approach to biomarker identification have yielded plasma proteins that demonstrate a noteworthy alteration in levels in AD patients when compared with controls. One study uncovered that alpha-2-macroglobulin and complement factor H, which are both evident in senile plaques, are present in elevated levels in AD plasma, with the latter only evident in increased levels for AD and not other types of dementia [37].

Phosphorylated tau protein in CSF is a new advancement in the search for AD biomarkers. The concentration of phosphorylated tau directly correlates with the state of tau in the brain. Unlike tau, concentrations of phosphorylated tau does not increase after a stroke or any other diseases, making phosphorylated tau a useful biomarker for AD. Phosphorylated tau protein in CSF has been seen to have high specificity, relative to tau protein for AD. Furthermore, other diseases, such as Parkinson's and depression, have normal concentrations of tau in individuals. Therefore, the specificity of phosporylated protein will prove useful since it will be able to distinguish AD from other types of dementias [1].

 In a study focusing on CSF biomarkers and incipient AD, the researchers concluded that combining tau and A*β*42 as a biomarker had an 83% specificity for detection of AD, while combining tau and phosphorylated tau as a biomarker yielded in a slightly higher specificity. Furthermore, T-tau, (what is this? Total tau), P-tau (what is this? Phosph tau), and A*β*42 have been proven to be strong markers for the development of AD in patients with mild cognitive impairment (MCI). If proven by other studies as well, this result could have an enormous impact on the design of clinical trials of patients with MCI. As convincing as these results may seem, more studies are still required to discover which combination of potential biomarkers generates the highest specificity. To increase specificity, a longer follow-up time (preferably more than five years) is required, because some cases in this study could have developed AD after the study had completed. Another useful technique in identifying incipient AD is through neuroimaging methods and cognitive tests. The downfall to these method is that each is correlated with disease severity. Trying to detect incipient AD during the earlier stages would increase the overlap between patients who actually have incipient AD and patients who have other illnesses [38].

Another potential biomarker that is present in elevated levels in AD patients is alpha-1-antitrypsin (A1AT), which can also be found in senile plaques and neurofibrillary tangles. A1AT is a serine protease inhibitor that is responsible for restraining overexpressed proteases during inflammation. Therefore, when it is oxidized to its precursor form and unable to perform this task, there is the characteristic inflammation seen in AD pathology [39]. Another protein associated with the systemic inflammation observed in the AD patients is the elevated presence of alpha-1-antichymotrypsin (A1ACT), which is also a serine protease inhibitor. Increases in A1ACT levels have been shown to have a correlative relationship with the severity of pathology and have also been known to induce hyperphosphorylation of tau in neurons [40].

The search for individual plasma biomarkers has yet to yield a definitive candidate for the diagnosis of AD. However, there is the possibility of using multiple protein markers concurrently to identify the risk for disease. A recent pilot study demonstrated the potential of 18 different signaling proteins found in the plasma that could be used as a diagnostic tool. The study observed the alterations in 18 signaling proteins that could indicate changes in the periphery or central nervous system that are closely linked to Alzheimer's disease [41]. When a study was conducted to reproduce AD diagnosis using the 18 analyte panel, the attempt was unable to produce similar results. The study was able to indicate that a full 89-analyte panel might be useful in diagnosis when used concurrently with other predictive markers such as A*β* [42].

The utility of these and other plasma proteins as diagnostic biomarkers is evident through their accessibility and ability to indicate several pathological processes that are characteristically seen in AD. The differences in levels of plasma proteins between AD patients and controls does aid in the explanation of disease proliferation, but there needs to be further work done in this area to definitively demonstrate the diagnostic efficacy and reproducibility of these findings. Additionally, the requirements of a biomarker to be able to serve as a diagnostic tool as well as to determine disease progression and the effects of treatment make it more realistic to work towards establishing a coordinative plasma biomarker that relies on a combination of different markers to establish AD prognosis and treatment.

## 5. Epigenetics

Epigenetics is another field of study that relies primarily on the epigenetic regulation of pathology in AD to help elucidate potential biomarker candidates for the disease. The term epigenetics refers to the dynamic regulation in genomic functions that occur independently of DNA sequence and the modification of DNA and chromatin that leads to key characteristic aberrations of the disease. Abnormalities in the amyloid precursor protein, A*β*, and the hyperphosphorylation are implicated in the pathogenesis of AD, and it is plausible that alterations in these genes contribute to the pathways of the disease. By altering the structure of chromatin, and thereby the transcription and expression of the genes, epigenetic processes are capable of altering cellular function. The primary targets of epigenetic regulation are methylation and histone modification of the chromatin; therefore, technologies to determine DNA methylation and histone modification profiles could prove particularly useful in determining genetic variations and genes responsible for the proliferation of AD. Additionally, critical changes are projected to be in epigenetic structures occurring during progression of the disease, leading to significant alterations in the molecular structure of several cells, tissues, and organs.

The transmembrane protein amyloid precursor protein (APP) has been extensively investigated through an epigenetics perspective. There are several studies that support the notion that the abnormalities of epigenetic mechanisms could affect the expression of APP, which plays an essential role in controlling A*β* synthesis and formation of plaques. An earlier study showed that the APP gene was in fact controlled by methylation and determined that variations in methylation-induced APP expression in different parts of the brain and other tissues. The determination that alterations in the methylation of the APP gene directly influence its expression in a region-specific manner suggests that the changes seen in AD could be impacted by epigenetics. 

The role of DNA methylation in AD proliferation has also been studied through the analysis of human postmortem brain tissues and the methylation status of various promoters of genes that are closely linked to the pathology of AD. One study of the human cerebral cortex demonstrated an elevation in the methylation of the SORBS3 gene and a decrease in the methylation of S100A2 gene [43]. The former is responsible for encoding a cell adhesion molecule that is seen in neurons and glia, while the latter is a calcium-binding protein. While these alterations in methylation status are normally seen in nondemented aging, the shift was much more evident in AD patients. Another study demonstrated that the promoter regions of the apolipoprotein E (APOE) and (MTHFR) genes were hypermethylated in AD patients in comparison to normal controls [44]. These and other studies demonstrate the notion that abnormal methylation of genes could certainly have a pronounced effect in AD. 

The aberrations in methylation and other epigenetic changes seen in AD demonstrate the need to further investigate this approach to AD pathology in order to elucidate the function of epigenetic regulation in this disease.

## 6. Metabolomics

One of the more novel approaches to discovering a diagnostic biomarker for Alzheimer's is the study of metabolomics, which utilizes the science behind biochemistry to detect any metabolic disruptions by simultaneously monitoring activity of various metabolites. Any unusual disturbances to activity in the metabolic network could be useful to better understanding the mechanisms of the disease. Although there has yet to be conclusive evidence to illustrate the existence of a metabolomic fingerprint that could serve as a conclusive diagnostic biomarker, this new field is able to make significant progress by creating a comprehensive map of metabolic pathway regulations that are influenced by genes and the environment. 

A recent pilot study probed the viability of utilizing this technology to better understand mechanistic pathways and possibly distinguish candidate biomarkers that could undergo further inquiry in the future. The study used postmortem samples of cerebrospinal fluid to attempt to discover any alterations in the metabolic pathways of AD patients and nondemented subjects. There were significant difference changes of tyrosine, norepinephrine tryptophan, purine, and tocopherol pathways in the AD samples when compared to controls [40]. Since the primary aim was to establish the practicability of this field and its potential to elucidate biochemical alterations of interest, there have yet to be any conclusive biomarkers yielded through this approach. Additionally, the study was performed on cerebrospinal fluid, but peripheral metabolomic signatures for AD compared to controls and other disease has not yet been explored. However, this form of exhaustive biochemical analysis could establish unique perspective on the pathways that are modified in disorders like AD that could further ascertain useful diagnostic markers.

## 7. Conclusion

The neurodegenerative pathology of Alzheimer's disease is the cause for the most prominent form of dementia and affects millions of people worldwide. While there are imaging and CSF-based technologies for the detection of this disease, it is important to inquire into other peripheral biomarkers that could offer a diagnosis that is both noninvasive and inexpensive. 

Our review has shown that a wide variety of peripheral biomarkers have been examined. Although all are easily obtained, they vary in their ability to detect already diagnosed disease. We suggest that biomarkers that are less able to detect already diagnosed disease with minimal error are not promising candidates for early detection of disease. In view of the promise of these selected peripheral biomarkers, we suggest that effort be devoted to determining their efficacy in large number of persons from a variety of populations. In addition, it is also essential that peripheral biomarkers that offer promise in terms of their ability to detect already diagnosed disease in large populations need to additionally demonstrate their ability to predict future diagnosis of AD by sufficient number of years to allow effective intervention. 

Establishing the utility of a peripheral biomarker may be considered in two phases. In Phase 1, it will be necessary to establish that the biomarker under consideration can detect already diagnosed AD. In Phase II, it will be necessary to demonstrate the ability of the biomarker to detect disease well in advance of the appearance of the current criteria for a diagnosis of AD.

The approaches reviewed offer important insight into the groundwork that has been established towards better comprehending the disease as well as newer fields of investigation that offer promising possibilities. These peripheral biomarkers not only offer the potential to establish diagnostic tools for clinical use, but also lay the foundation for better understanding the mechanisms of the disease that could reveal methods for the treatment and even the prevention of AD.

##  Funding

This paper was funded by NIAP30 AG 019610 and the Banner Sun Health Research Institute.
